# Supplementary material for: Dynamicasome—a molecular dynamics-guided and AI-driven pathogenicity prediction catalogue for all genetic mutations
Source: Commun Biol. 2025 Jul 7;8:958. doi: 10.1038/s42003-025-08334-y (PMC12234709; doi:10.1038/s42003-025-08334-y)
Supplement: Supplementary file 1 — Supplemental Material [file 42003_2025_8334_MOESM1_ESM.pdf]

SUPPLEMENTAL MATERIAL

"Dynamicasome -- A molecular dynamics-guided and AI-driven pathogenicity prediction catalogue for all genetic mutations"

Naeyma N. Islam[1], PhD; Mathew A. Coban[1]; Jessica M. Fuller[1]; Caleb Weber[1]; Rohit Chitale[2]; Benjamin Jussila[3], Trisha J. Brock, PhD[3]; Cui Tao[4]; Thomas R. Caulfield, PhD[1,4-9,10*]

[1] Department of Neuroscience, Mayo Clinic, Jacksonville FL USA
[2] Department of Infectious Disease, Mayo Clinic, Jacksonville FL USA
[3] InVivo Biosystems, Inc., Eugene, Oregon 97402, USA
[4] Department ofArtificial Intelligence and Informatics, Mayo Clinic, Jacksonville FL USA
[5] Quantitative Health Sciences, Biostatistics Division, Mayo Clinic, Jacksonville FL USA
[6] Department of Biochemistry and Molecular Biology, Mayo Clinic, Rochester MN USA
[7] Department of Neurosurgery, Mayo Clinic, Jacksonville FL USA
[8] Department of Cancer Biology, Mayo Clinic, Jacksonville FL USA
[9] Department of Clinical Genomics, Mayo Clinic, Rochester MN USA
[10] Digital Ether Systems and Computing, Inc, Miami, FL USA

*Corresponding Author: Thomas R. Caulfield, PhD
Email: thomas@digitalethercomputing.com

Supplemental Figures and Data.

Schematic S1. Overview of the Gene-to-Protein through Data Harvesting and AI Differentiation.

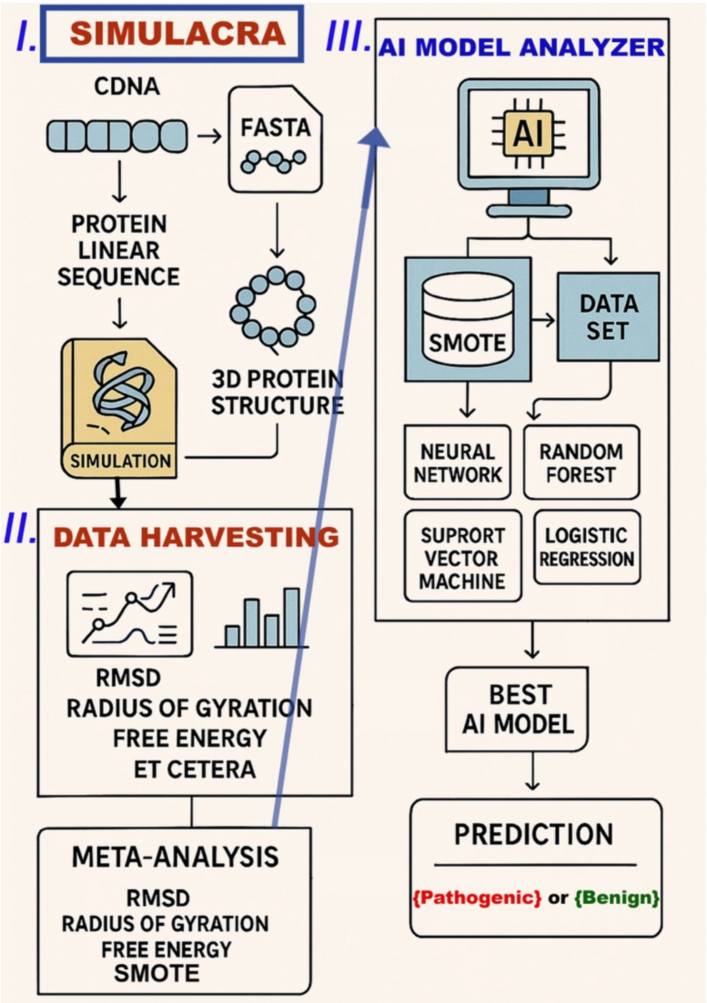

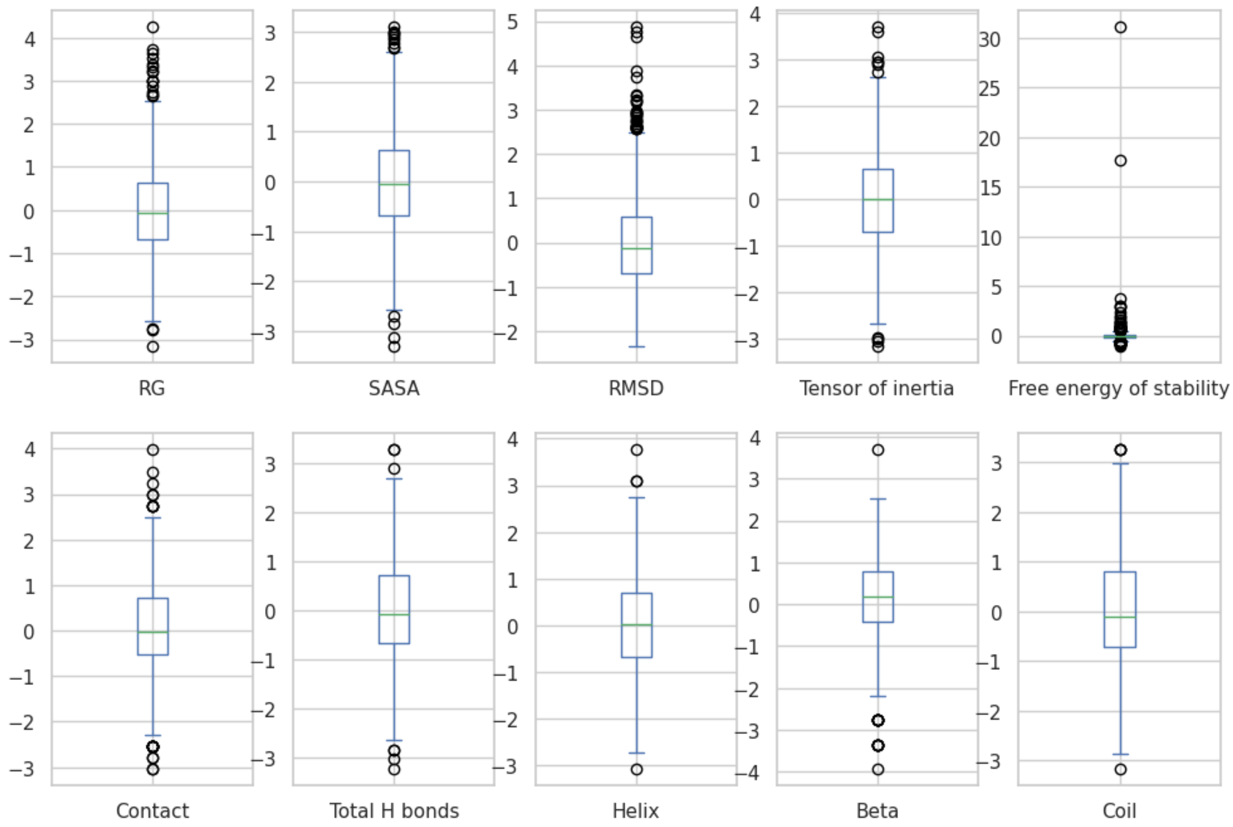

Fig. S1. MDS-extracted features show varied distributions across PMM2 mutations. Box plots of the distribution of indicated MDS-extracted features across all PMM2 missense mutations. Each plot depicts the range as a line, the interquartile range as a box, the median as a horizontal line inside the box, and outliers as circles. “Contact” refers to all amino acid contacts; “H bonds” refers to hydrogen bonds; “Helix”, “Beta”, and “Coil” refer to secondary structures.

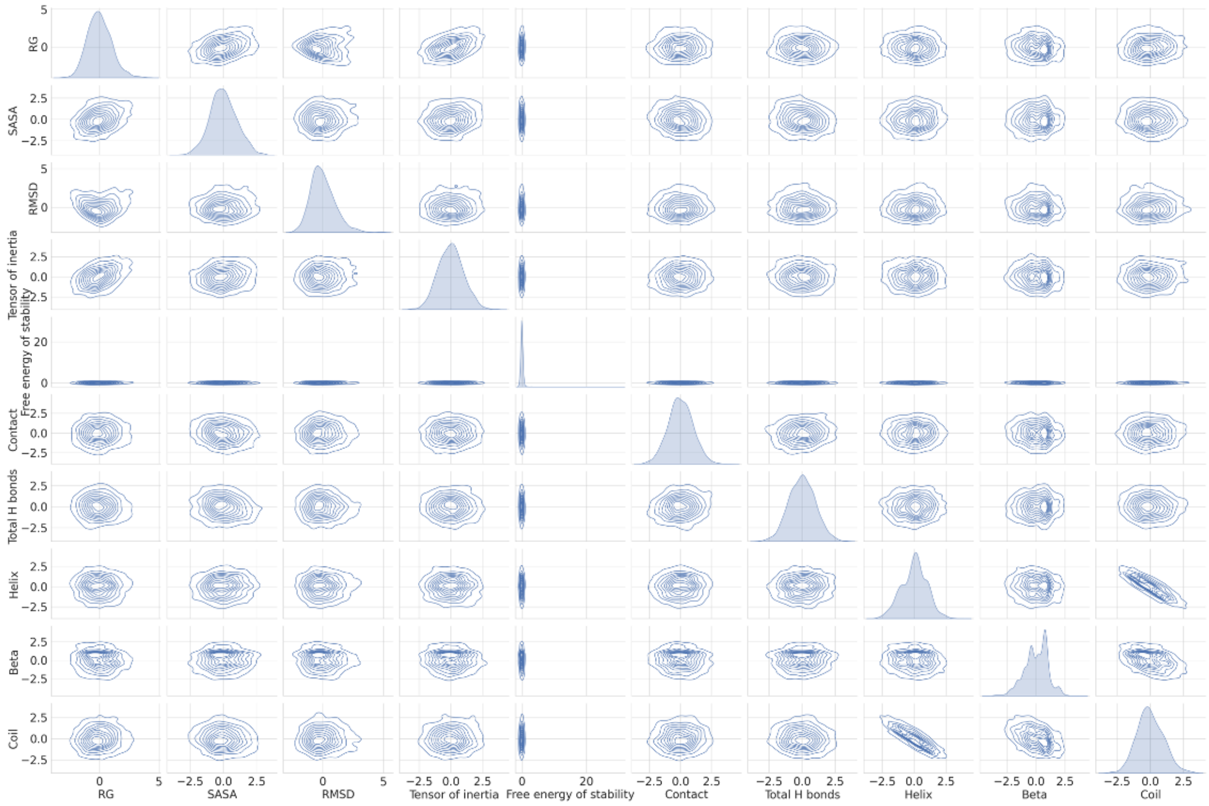

Fig. S2. Pair plots reveal correlations between MDS-extracted features of PMM2 variants. Scaled readouts of each feature extracted from MDS of PMM2 variants are plotted against each other to reveal correlations.

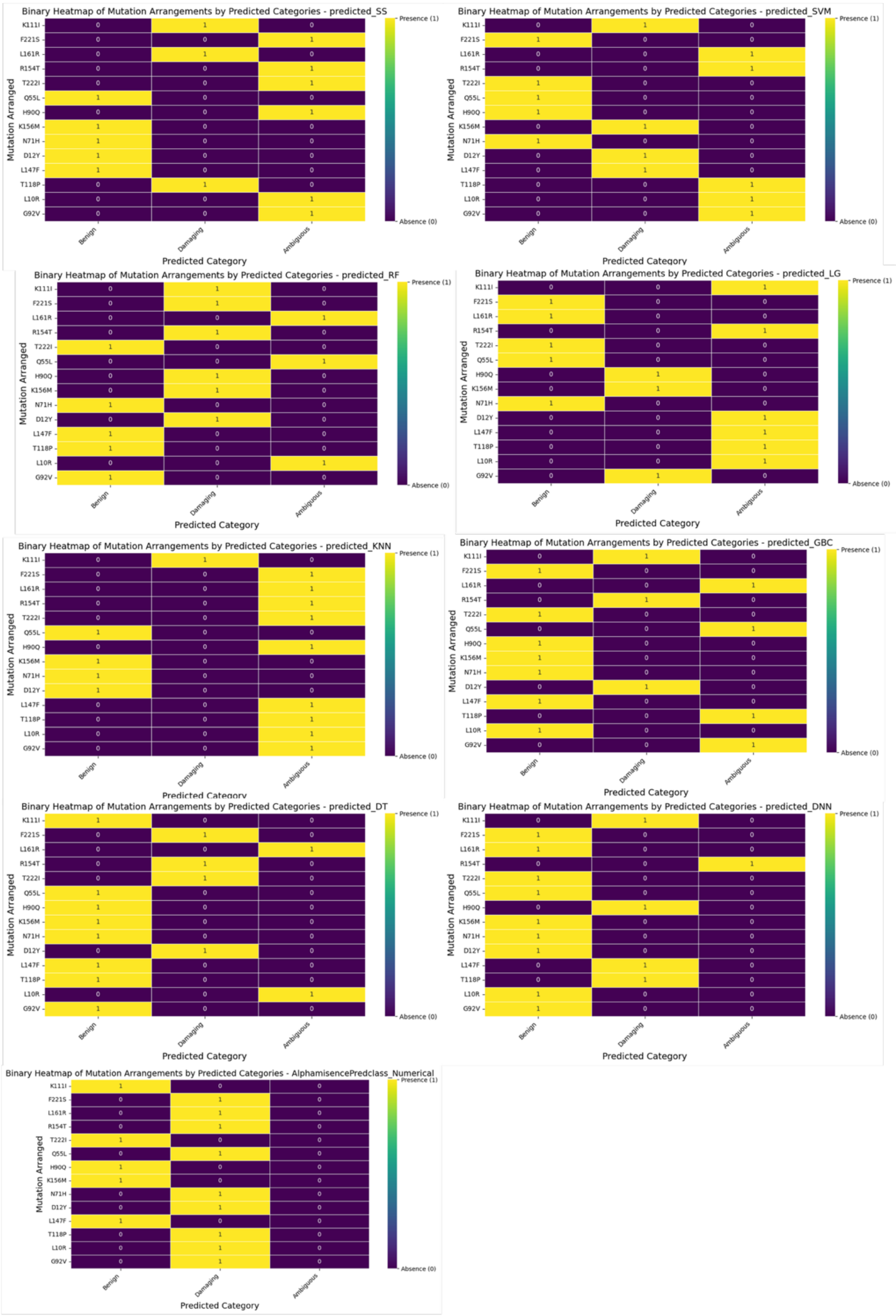

Fig. S3. Advanced AI models show variations in their predictions for individual PMM2 mutations. Binary heat maps display how indicated advanced AI models called a subset of

PMM2 mutations of unknown significance. Yellow denotes the category called by the model (benign, damaging, or ambiguous), while purple denotes the other two categories not called.

Table S1. Clinical significance of all labeled PMM2 missense variants in ClinVar.

aa in WT PMM2	Residue Number in PMM2	aa post-mutation	ClinVar Classification	Second ClinVar Classification
C	9	F	LP	
C	9	Y	P	LP
T	18	S	P	LP
R	21	G	P	
L	32	V	LP	
L	32	R	P	
Q	37	L	B	LB
V	43	M	LP	
V	44	A	P	LP
D	65	Y	P	
P	69	T	LP	
N	101	K	P	
L	104	V	P	
A	108	V	P	LP
P	113	L	P	
P	113	T	LP	
G	117	R	LP	
F	119	L	P	
I	120	M	LP	
I	120	T	P	LP
R	123	Q	P	LP
V	129	L	P	
V	129	M	P	LP
P	131	A	P	LP
I	132	T	P	
I	132	F	P	
E	139	K	P	LP
R	141	H	P	LP
F	144	C	LP	
F	144	V	LP	
F	144	L	P	LP
D	148	N	P	LP
I	153	T	P	LP
F	157	C	LP	

F	157	S	P	LP
R	162	P	LP	
R	162	W	P	LP
G	176	V	LP	
G	176	S	LP	
F	183	S	P	
G	186	R	P	
D	188	E	LP	
D	188	G	P	LP
D	188	Y	LP	
H	195	R	LP	
E	197	A	B	LB
F	207	S	P	LP
G	208	A	P	LP
N	216	I	P	LP
H	218	D	LP	
D	223	E	P	
D	223	N	P	
T	226	S	P	LP
G	228	C	P	LP
V	231	M	P	
T	237	M	P	LP
T	237	R	P	LP
R	238	P	P	LP
R	239	S	LP	
R	239	W	P	
C	241	W	LP	
C	241	S	P	

aa: amino acid; P: pathogenic; LP: likely pathogenic; LB: likely benign; B: benign. Note: some mutations were submitted to ClinVar multiple times, leading to a second classification of pathogenicity in some instances. If these classifications conflicted, the mutation was considered of uncertain significance. Mutations labeled as uncertain were omitted from this table.

Table S2. **Allele frequency, heterozygosity, and ClinVar clinical significance of PMM2 variants in gnomAD deemed benign by our evaluation.**

**aa in WT PMM2**	**Residue Number in PMM2**	**aa Post-mutation**	**ClinVar Classification**	**Second ClinVar Classification**	**Allele Frequency**	**Number of Homozygotes in gnomAD**
E	197	A	B	LB	0.023	535
Q	37	L	B	LB	0.000549	16
A	228	V	LB		0.000902	8
D	30	E	VUS		0.00038	8
R	238	C	VUS		0.000556	8
M	212	V	VUS		0.000473	4
M	227	T	VUS		0.0000116	2
V	196	M	VUS		0.0000279	1
E	219	D	VUS		0.0000756	1
V	182	I	-		0.0000116	1
H	221	Q	-		0.0000716	1

aa: amino acid; LB: likely benign; B: benign; VUS: variant of unknown significance.

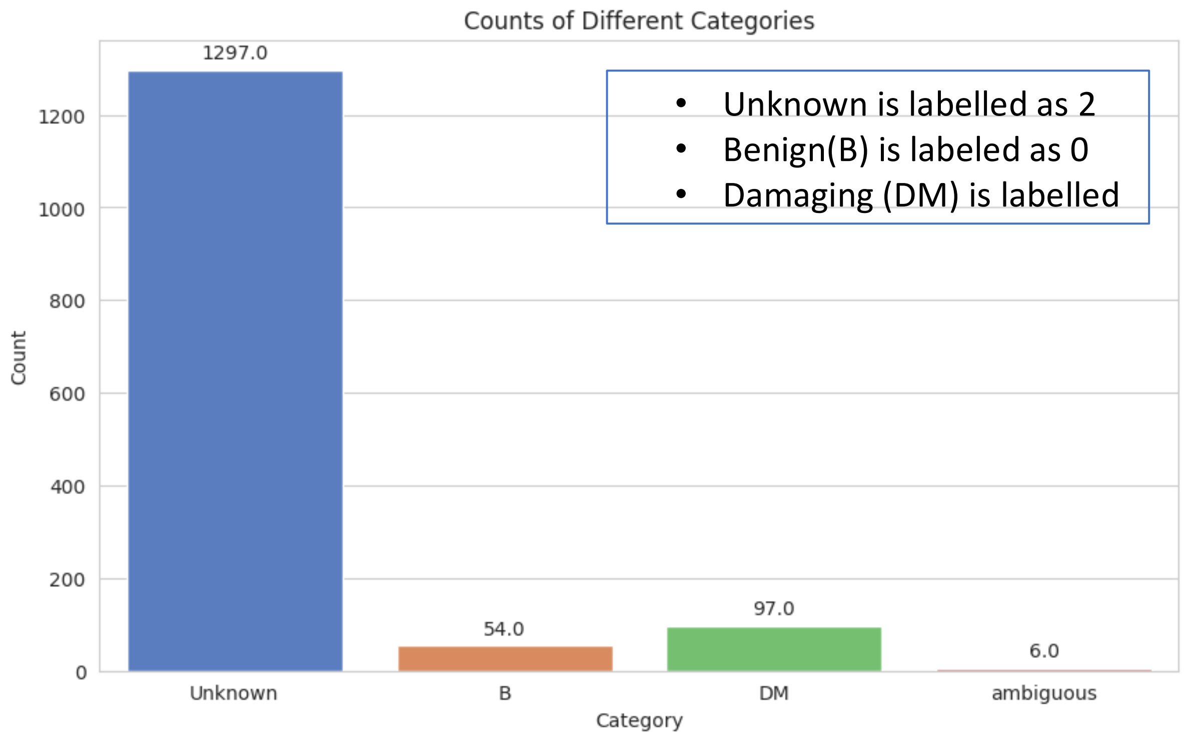

Fig. S4. Distribution of PMM2 missense mutations categorized by clinical annotation. The bar plot shows the number of mutations in each category as obtained from human clinical datasets. Mutations are labeled as Unknown (2)**,** Benign (0)**,** Damaging (1)**, and** Ambiguous (3)**.** The majority of mutations (n = 1297) are clinically uncharacterized (unknown), while 54 are benign, 97 are damaging, and 6 are labeled as ambiguous or of uncertain significance. These labels were used for supervised learning in the model training phase

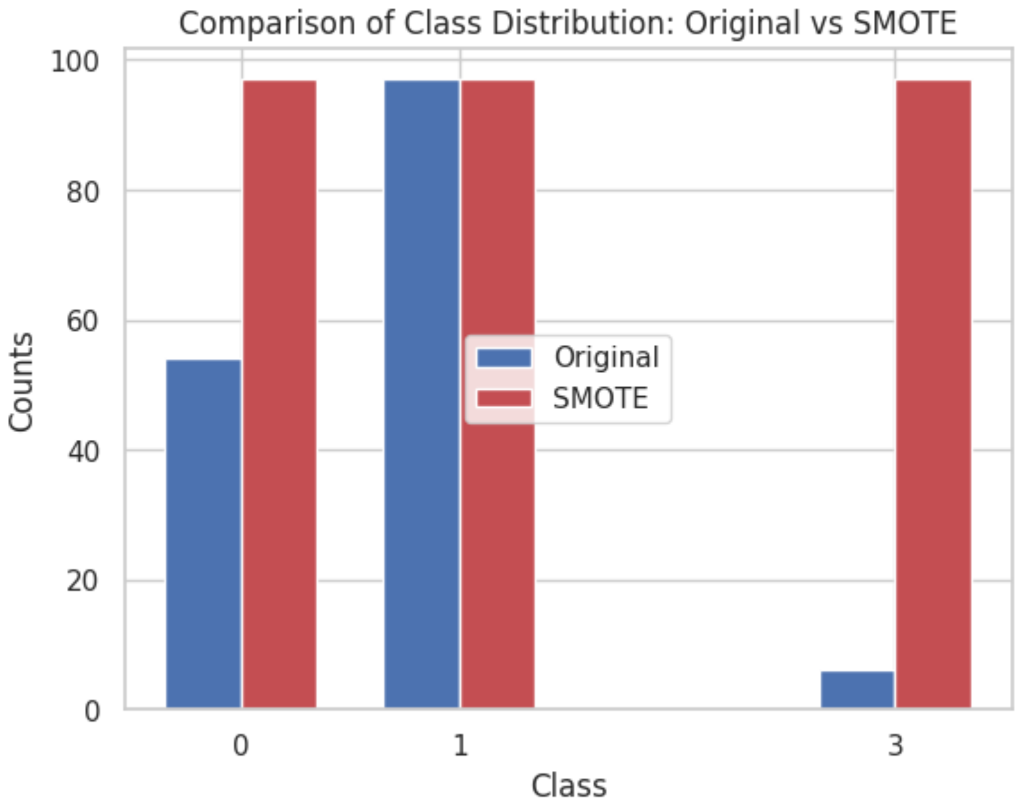

Fig. S5. Comparison of class distribution before and after SMOTE balancing. The plot illustrates the distribution of labeled PMM2 mutations across three classes—Benign (0), Damaging (1), and Ambiguous (3)—in the original dataset (blue bars) and after applying SMOTE (Synthetic Minority Over-sampling Technique; red bars). SMOTE was applied only to the training data to address class imbalance by oversampling the minority classes (Benign and Ambiguous) to match the number of samples in the majority class (Damaging), enabling more balanced model training

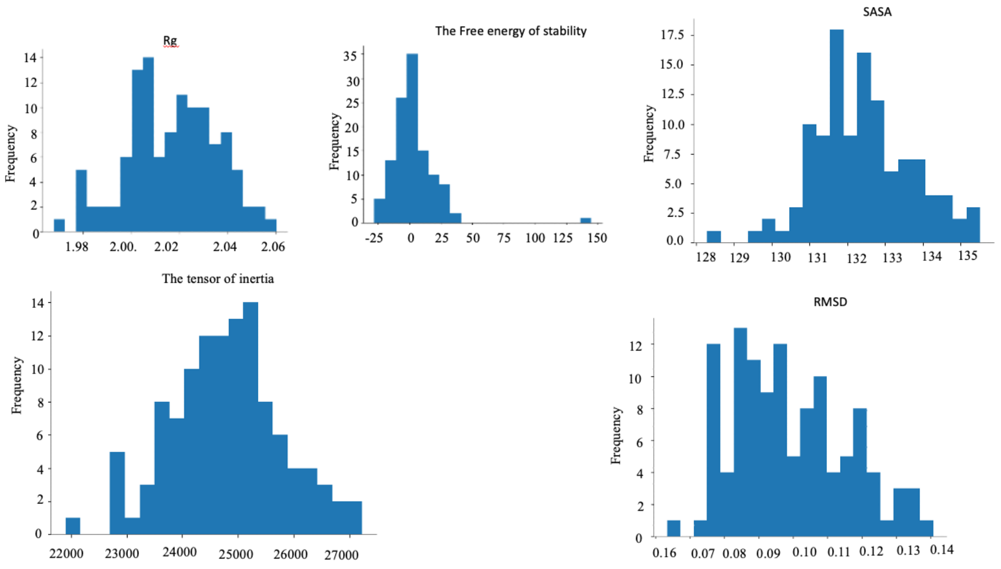

Fig. S6. Distribution of key molecular dynamics (MD)-derived features across all simulated PMM2 variants. Histograms represent the frequency of five biophysical properties calculated from MD trajectories: Radius of gyration (Rg), Free energy of stability, Solvent Accessible Surface Area (SASA), Tensor of inertia**,** and Root Mean Square Deviation (RMSD). These features capture conformational and energetic changes in the protein structure induced by mutations and were used as input variables for model training. The plotted values represent the average across frames in the simulation trajectories for each variant.
