# Supplementary material for: Dynamicasome—a molecular dynamics-guided and AI-driven pathogenicity prediction catalogue for all genetic mutations
Source: Commun Biol. 2025 Jul 7;8:958. doi: 10.1038/s42003-025-08334-y (PMC12234709; doi:10.1038/s42003-025-08334-y)
Supplement: Supplementary file 2 — Description of Additional Supplementary Data Files [file 42003_2025_8334_MOESM2_ESM.docx]

**Supplentary Data 1.**

Naeyma et al., **“Dynamicasome -- A molecular dynamics-guided and AI-driven pathogenicity prediction catalogue for all genetic mutations”**

**Description of Additional Supplementary File.**

1. Balanced_SMOTE_X_Fetures.xlsx
   1. Here is the balance dataset containing the features by SMOTE algorithm
2. Balanced_SMOTE_Y_Labels.xlsx
   1. Here is the balance dataset containing the labels by SMOTE algorithm
3. Latest_AllModel_WithOutSide_Program_May10th_2024.xlsx
   1. Here is the metadata for all calculated and benchmark model
4. MutR_10ScaledF_cata_NumCata.xlsx
   1. Here is all the scaled feature data with their numerical label
5. Decission_Tree_Model_SignificantFeatureMay10th.ipynb
   1. Here is the code for the model training
6. DNN_Model_May9th.ipynb
   1. Here is the DNN python code used
7. KNN_Model_May10th.ipynb
   1. Here is the KNN python code used
8. Logistic_Regression_Model_May10th.ipynb
   1. Here is the Log Regress python code used
9. RF_Model_May9th.ipynb
   1. Here is the RF python code used
10. SemiSupervised_may10th.ipynb
    1. Here is the semisupervised python code used
11. SVM_Model_May10th.ipynb
    1. Here is the SVM python code used
